# Effect of the categorization method on the diagnostic performance of ultrasound risk stratification systems for thyroid nodules

**DOI:** 10.3389/fonc.2023.1073891

**Published:** 2023-04-25

**Authors:** Chao Fu, Yiyang Cui, Jing Li, Jing Yu, Yan Wang, Caifeng Si, Kefei Cui

**Affiliations:** ^1^ Department of Ultrasound, The First Affiliated Hospital of Zhengzhou University, Zhengzhou, China; ^2^ Department of Interventional Radiology, The First Affiliated Hospital of Zhengzhou University, Zhengzhou, China

**Keywords:** thyroid neoplasm, thyroid nodule, ultrasonography, surgical histology, thyroid imaging reporting and data system

## Abstract

**Objective:**

To evaluate whether the categorization methods of risk stratification systems (RSSs) is a decisive factor that influenced the diagnostic performances and unnecessary FNA rates in order to choose optimal RSS for the management of thyroid nodules.

**Methods:**

From July 2013 to January 2019, 2667 patients with 3944 thyroid nodules had undergone pathological diagnosis after thyroidectomy and/or US-guided FNA. US categories were assigned according to the six RSSs. The diagnostic performances and unnecessary FNA rates were calculated and compared according to the US-based final assessment categories and the unified size thresholds for biopsy proposed by ACR-TIRADS, respectively.

**Results:**

A total of 1781 (45.2%) thyroid nodules were diagnosed as malignant after thyroidectomy or biopsy. Significantly lowest specificity and accuracy, along with the highest unnecessary FNA rates were seen in EU-TIRADS for both US categories (47.9%, 70.2%, and 39.4%, respectively, all *P* < 0.05) and indications for FNA (54.2%, 50.0%, and 55.4%, respectively, all *P* < 0.05). Diagnostic performances for US-based final assessment categories exhibited similar accuracy for AI-TIRADS, Kwak-TIRADS, C-TIRADS, and ATA guidelines (78.0%, 77.8%, 77.9%, and 76.3%, respectively, all *P* > 0.05), while the lowest unnecessary FNA rate was seen in C-TIRADS (30.9%) and without significant differences to that of AI-TIRADS, Kwak-TIRADS, and ATA guideline (31.5%, 31.7%, and 33.6%, respectively, all *P* > 0.05). Diagnostic performance for US-FNA indications showed similar accuracy for ACR-TIRADS, Kwak-TIRADS, C-TIRADS and ATA guidelines (58.0%, 59.7%, 58.7%, and 57.1%, respectively, all *P* > 0.05). The highest accuracy and lowest unnecessary FNA rate were seen in AI-TIRADS (61.9%, 38.6%) and without significant differences to that of Kwak-TIRADS(59.7%, 42.9%) and C-TIRADS 58.7%, 43.9%, all *P* > 0.05).

**Conclusion:**

The different US categorization methods used by each RSS were not determinant influential factors in diagnostic performance and unnecessary FNA rate. For daily clinical practice, the score-based counting RSS was an optimal choice.

## 1 Introduction

Thyroid nodules occur in about 20%-70% of the adult population with a wide use of imaging modalities and the incidence increases with age (1, 2). Ultrasound (US)-based risk stratification systems (RSSs) play an essential role in reducing unnecessary nodule biopsies and require an appropriate sensitivity for thyroid malignancy (3). Recent comparative studies showed a wide spectrum of diagnostic performance for the category-based and biopsy criteria (4–10), which render the interpretation of study results from different societies difficult and reduce the effectiveness of communication with health care professionals in other areas. Thus, it is necessary to analyze the influencing factors of the different diagnostic performances among the RSSs to provide a basis for the selection of the optimal RSS in daily clinical practice.

To manage thyroid nodules, the various RSSs used different US features and even different size thresholds for fine-needle aspiration (FNA) (2, 11–16). Recent evidence suggests that the size thresholds for FNA in each RSS influence diagnostic performances and unnecessary FNA rates (17–19). Based on the way US features are utilized in the categorization method, RSSs can be broadly divided into two types: pattern-based RSSs and score-based RSSs, the latter of which can be calculated by the weighting method and the counting method. To the best of our knowledge, no systematic study has examined whether diagnostic performances are affected by the categorization method based on US features. Previous research comparing score-based and pattern-based RSSs has found the two categorization methods for RSSs had their own peculiarity (20). However, that study failed to take into account of the potential impact of the size threshold for FNA.

The objective of this research is to examine whether how US features are utilized (pattern-based, score-based counting, and score-based weighting) has an impact on the diagnostic performance and unnecessary FNA rates using six RSSs: the EU-TIRADS (European Thyroid Radiology) (14), ATA guideline (American Thyroid Association) (2), C-TIRADS (Chinese Thyroid Imaging Reporting and Data System) (16), Kwak-TIRADS (which was issued by Kwak et al.) (13), ACR-TIRADS (American College of Radiology) (11), and AI-TIRADS (which was a simplified version of ACR TIRADS by artificial intelligence algorithm) (21). Therefore, by using US-based final assessment categories and the uniform size thresholds for FNA suggested by ACR-TIRADS, respectively, we compared the diagnostic performance and unnecessary FNA rates of the six RSSs.

## 2 Materials and methods

### 2.1 Study cohort

The Scientific Research and Clinical Trials Ethics Committee of the First Affiliated Hospital of Zhengzhou University of China approved this retrospective study and granted a waiver of written informed consent for use of data. From July 2013 to January 2019, a consecutive of 2744 patients with 4075 thyroid nodules underwent thyroid US examinations and thyroidectomy or US-FNA at our institution, a tertiary referral center. 131 nodules in 77 patients were excluded from this study because the US images were blurred or lacked two vertical sections or because they had a lack of definitive cytopathologic results after performing US-FNA without surgical confirmation. Finally, a total of 3944 nodules in 2667 patients were included in this study (2045 women and 622 men). A total of 3591 nodules underwent thyroidectomy and 353 nodules underwent US-FNA. Mean age of the patients was 47.2 years ±12.2 (range, 7 - 82 years). Mean size of the 3944 thyroid nodules was 16.8 mm ± 14.6 (range, 1.5 - 102.0mm).

### 2.2 US examinations and imaging analysis

All US examinations were performed with a 5-14-MHz linear probe and a real-time US system (TOSHIBA Aplio300). US examinations were performed by a senior radiologist (K.F.C) with 33 years of experience in thyroid imaging. All the US examinations complied with the AIUM (22) protocol for thyroid and parathyroid scanning. During the US examination, images of each target nodule(thyroid nodules and suspicious cervical lymph nodes) generally obtained were at least on gray scale and with one Doppler US image in each transverse and longitudinal plane. Additional images were obtained to substantiate the importance of the US features of the nodules. The US data were recorded and stored in the internal hard-disk for further offline analysis. The nodule’s size was defined by the maximal diameter at US.

ACR-TIRADS (11), C-TIRADS (16), and EU-TIRADS (14) have their own US lexicon for describing thyroid nodules, but ATA guidelines and Kwak TIRADS do not. It should be mentioned that AI-TIRADS is a simplified version of ACR-TIRADS which shares the definition of ACR-TIRADS’s US lexicon of thyroid nodules. That is to say, the definitions of US lexicons among the ACR-TIRADS, C-TIRADS, and EU-TIRADS, which should be well-defined and simple to use in clinical application, largely overlap. Therefore in this study, the selection of the different US lexicons is based on the principle of simplicity and accuracy of definitions. For instance, punctate echogenic foci/microcalcification: it having no posterior acoustic posterior artifacts (11); macrocalcification: it having posterior acoustic posterior artifacts (11); peripheral calcifications: echogenic foci are located at the periphery of the nodules, and might appear as a continuous or discontinuous ring or arc involving more than a third of the margin (16); orientation (shape): it is suggested to judge orientation (shape) on the basis of accurate measurement, but the visual evaluation is also acceptable (16). Meanwhile, orientation (shape) is not limited to transverse or longitudinal sections (16, 23).

An overview and a discussion session were held by a senior radiologist (K.C.) with 33 years of experience in thyroid imaging to establish consensus regarding the definitions of the US lexicons from the ACR-TIRADS, C-TIRADS, and EU-TIRADS, including size (the maximal diameter at US), composition (solid, predominately solid, predominately cystic, cystic, spongiform), echogenicity (hyperechoic, isoechoic, hypoechoic, markedly hypoechoic), orientation (vertical/taller-than-wide, horizontal/wider-than-tall), margins (smooth, irregular, lobulated, ill-defined, extrathyroidal extension), echogenic foci (punctate echogenic foci, macrocalcification, peripheral calcifications, comet-tail artifacts). Subsequently, an interactive case-based training session was conducted by using 30 representative thyroid nodules not included in this study.

Finally, US features were independently reviewed by two radiologists (C.F and Y.J.H, with 13 and 12 years, respectively, of clinical experience performing thyroid US scans and evaluating thyroid US images) blinded to the biopsy results and the final pathological diagnoses. A reviewer (Y.Y.C.), who had no previous knowledge of the FNA results or surgical pathologies, classified nodules based on the assessed US features and determined the eligibility for FNA of each nodule based on the size and RSS category.

Isoechoic nodules with an irregular margin, microcalcification, and vertical orientation (taller-than-wide shape) were categorized as unclassified nodules in the ATA guidelines (8, 9), which were categorized as intermediate-suspicion nodules, based on previous studies (5, 24, 25).

### 2.3 Data and statistical analysis

In previous studies, the diagnostic performance can be calculated according to US-based final assessment categories (raw diagnostic performance, before applying size thresholds for FNA) (26, 27) and indications for FNA (diagnostic performance of RSSs after applying size thresholds for FNA) (17, 28).

In this study, the triage of the six RSSs was dichotomized into positive (category 4b and 5 for Kwak-TIRADS and C-TIRADS, category 4 and 5 for ACR-TIRADS, AI-TIRADS, EU-TIRADS, and ATA guideline) and negative (category 2 to 4a for Kwak-TIRADS and C-TIRADS, category 1 to 3 for ACR-TIRADS, AI-TIRADS, and ATA guideline, category 2 and 3 for EU-TIRADS) according to the level of suspicion each assessment category represents when calculating diagnostic performance. The dichotomy has been introduced in previous studies (20).

In order to rule out the influence of different size thresholds for FNA in each RSS, we used the uniform size thresholds proposed by ACR-TIRADS for the six RSSs according to the similar estimated malignancy rates in **Table 1** (17). The unnecessary FNA rate was calculated as the proportion of benign nodules in the nodules recommended for FNA. We evaluated the diagnostic performances using sensitivity, specificity, accuracy, AUC, and unnecessary FNA rate according to US-based final assessment categories and indications for FNA, respectively.

**Table 1:** The uniform size thresholds suggested by ACR-TIRADS for fine-needle aspiration in the six risk stratification systems.

	No FNA	FNA≥25mm	FNA≥15mm	FNA≥10mm
ACR-TIRADS	2-not suspicious	3-mildly suspicious	4-moderately suspicious	5-highly suspicious
AI-TIRADS	2-not suspicious	3-mildly suspicious	4-moderately suspicious	5-highly suspicious
Modified ATA guideline	Benign 2-verylow suspicion		3-low suspicion 4-intermediate suspicion	5-high suspicion
Modified EU-TIRADS	Benign	3-low risk	4-intermediate risk	5-high risk
Modified Kwak-TIRADS	3-no suspicious US feature	4a-one suspicious US feature	4b-two suspicious US feature	4c-three or four suspicious US feature 5-five suspicious US features
Modified C-TIRADS	3-no suspicious US feature	4a-one suspicious US feature	4b-two suspicious US feature	4c-three or four suspicious US feature 5-five suspicious US features

ACR-TIRADS, American College of Radiology Thyroid Imaging Reporting and Data System; AI-TIRADS, it is a simplified version of ACR-TIRADS by artificial intelligence algorithm; ATA guideline, American Thyroid Association guideline; EU-TIRADS, European Thyroid Radiology Thyroid Imaging Reporting and Data System; Kwak-TIRADS, Thyroid Imaging Reporting and Data System was issued by Kwak et al; C-TIRADS, Chinese Thyroid Imaging Reporting and Data System; The modified EU-TIRADS, ATA guideline, Kwak-TIRDS and C-TIRADS incorporated the same size thresholds suggested by the ACR-TIRADS.

The demographic data between benign and malignant nodules were compared by using the independent two-sample t-test for numerical data (age and nodule size) and the Chi-square test for categorical data (sex and size distribution). Sensitivity, specificity, accuracy, and unnecessary FNA rates among multiple groups (six RSSs) were determined using ANOVA followed by Bonferroni multiple comparison test. For multiple comparison, we provided false discovery rate adjusted p value. Areas under the receiver operating characteristic curve (AUCs) along with 95% CIs were calculated and compared using the DeLong method. Statistical data were performed with SPSS software for Windows (version 26.0, SPSS Institute, USA) and MedCalc software (version 18.2.1, Mariakerke, Belgium). Two-sided *P* values < 0.05 were considered to indicate statistical significance.

## 3 Results

### 3.1 Baseline clinicopathological characteristics

Demographics and US features of the patients and nodules are summarized in **Table 2**. Of 3944 thyroid nodules, 2163(54.8%) were benign and 1781(45.2%) were malignant. Papillary thyroid carcinomas were the most common malignant nodules [1719 papillary thyroid carcinomas (including 47 follicular variant thyroid carcinomas), 22 follicular carcinomas, 18 medullary carcinomas, 4 lymphomas, 2 anaplastic carcinomas, 1 metastasis, 8 squamous cell carcinoma, 4 mixed carcinomas, 2 Hürthle cell carcinomas, and 1 poorly differentiated carcinoma (insular carcinoma)]. Nodular goiters were the most common benign nodules [1696 nodular goiters, 37 follicular adenomas, 130 thyroiditis (including lymphocytic, subacute, and granulomatous), 193 adenomatous goiter, 12 Hürthle cell adenomas, 18 hemorrhagic cysts, 31 Graves’ diseases, 39 simple goiters, 1 cystic lymphangioma, 4 cysts, and 2 neurilemmomas].

**Table 2:** Summary of demographic features for the patients with thyroid nodules.

Characteristics	Final pathology		Total	*P-*Value
	Benign	Malignant		
No. of nodules	2163 (54.8)	1781 (45.2)	3944	
Age				0.000
Mean (years)	49.3 ± 12.1	44.7 ± 11.9	47.2 ± 12.2	
Range (years)	10 - 82	7 - 82	7 - 82	
<55 years	1454 (67.2)	1435 (80.6)	2889 (73.3)	0.000
≥55 years	709 (32.8)	346 (19.4)	1055 (26.7)	
Gender				0.131
Male	457(21.1)	444(24.9)	901(22.8)	
Female	1706(78.9)	1337(75.1)	3043(77.2)	
Size				0.000
Mean (mm)	20.2±15.8	13.0±11.6	16.9±14.5	
Range (mm)	2.0-100.0	1.5-102.0	1.5-102.0	
<10 mm	764 (35.3)	951 (53.4)	1715 (43.5)	0.000
10 - 20 mm	488 (22.6)	531 (29.8)	1019 (25.8)	
≥20 mm	911 (42.1)	299 (16.8)	1210 (30.7)	

Data in parentheses are percentages.

Gender was not significantly associated with malignancy risk (*P* > 0.05). Age under 55 years had exceptionally higher risk of malignancy compared with ≥ 55 years of age (*P* < 0.05). Benign thyroid nodules were significantly larger than malignant nodules (20.2 mm ± 15.8 vs. 13.2 mm ± 11.6, *P* < 0.05).

### 3.2 Malignancy rates and proportion of nodule numbers at each category

**Figure 1:**
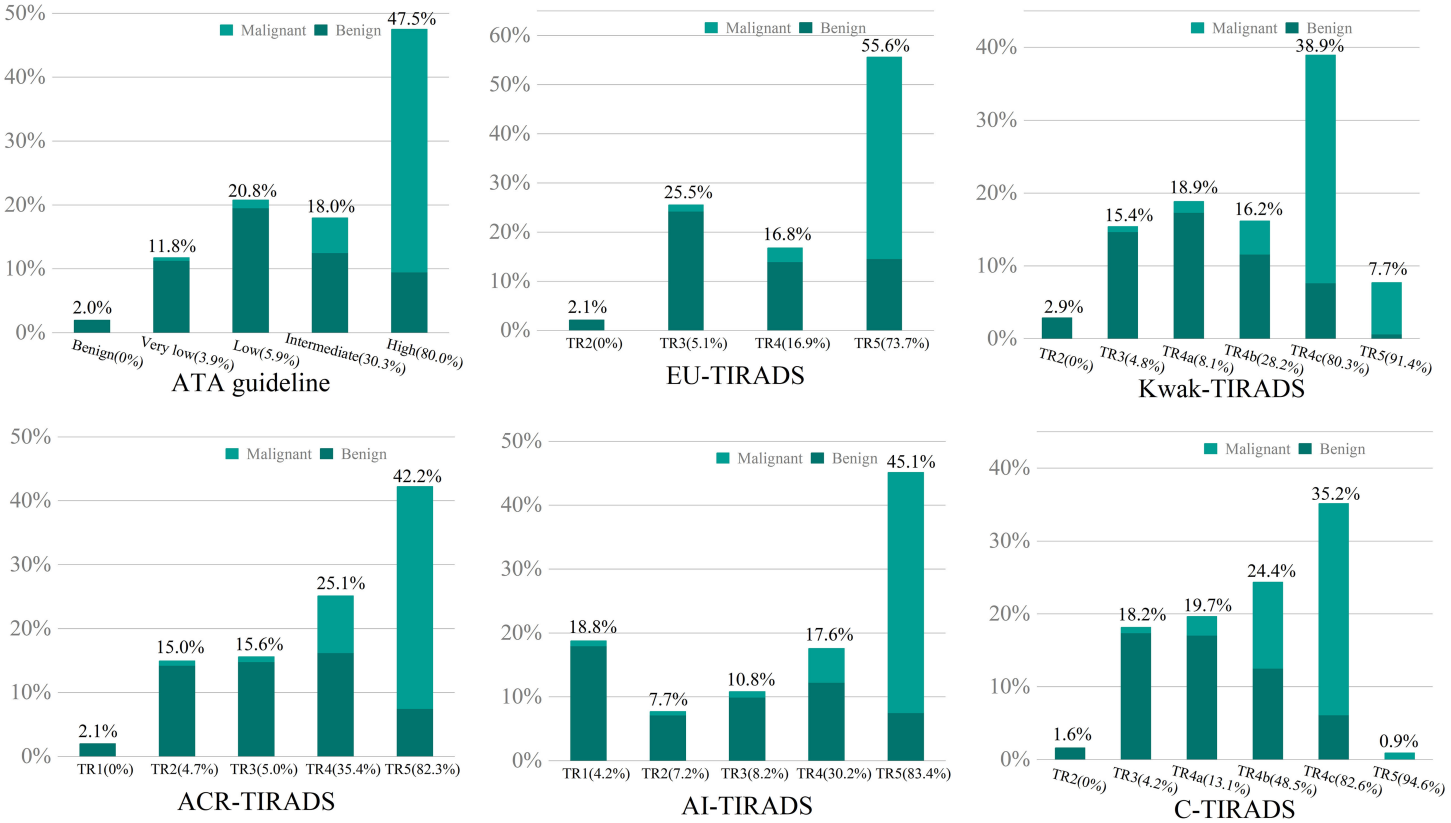
The calculated malignancy rates and proportion of nodule numbers at each category in the six RSSs were plotted on the bar graph with the abscissa as categories and the ordinate as proportion of number. Numbers in brackets depict the calculated malignancy rates in each category.

In this study group, the malignancy rate was 45.2% (1781 of 3944). The malignancy rates in each RSS differed significantly according to categories (*P* < 0.05 for all). Within each RSS, malignancy rates increased as the categories increased (**Figure 1**). The calculated malignancy risks of almost all categories were well matched with the suggested malignancy risk range in each RSSs. The highest proportion of nodule members within 5 category (TR5) or high suspicion was witnessed in ATA guidelines, EU-TIRADS, ACR-TIRADS, and AI-TIRADS. However, 4c category (TR4c) contained a greater proportion of nodules members in C-TIRADS and Kwak-TIRADS.

321 (8.1%) nodules have not been specified and categorized in ATA guidelines, the malignancy rate was 37.7% (121/321). The malignancy rate increased from 24.2% (94/388) to 30.3% (215/709) in the intermediate after including the unspecified nodules into the intermediate category of the ATA guidelines.

### 3.3 Comparison of the diagnostic performance before applying size thresholds of FNA (according to US-based final assessment categories)

**Table 3** summarizes the diagnostic performances of the three groups (pattern-based, score-based counting and score-based weighting method) according the US-based final assessment categories.

**Table 3:** Diagnostic performances according to the US-based final assessment and FNA thresholds.

Category	Sensitivity	Specificity	Accuracy	UFR	AUC
ACR-TIRADS					
US	96.7% (95.8 - 97.4)	56.8% (54.6 - 58.8)	74.8% (73.4 - 76.1)	35.2% (33.4 - 37.0)	0.869 (0.858 - 0.879)
FNA	41.9% (39.6 - 44.2)	71.3% (69.5 - 73.1)	58.0% (56.4 - 59.7)	45.4% (42.7 - 48.2)	0.566 (0.551 - 0.582)
AI-TIRADS					
US	95.1% (94.0 - 96.1)	63.9% (62.0 - 65.9)	78.0% (76.6 - 79.3)	31.5% (29.6 - 33.4)	0.883 (0.871 - 0.893)
FNA	42.3% (40.1 - 44.6)	78.1% (76.4 - 79.8)	61.9% (60.4 - 63.5)	38.6% (36.0 - 41.3)	0.602 (0.587 - 0.617)
Kwak-TIRADS					
US	95.0% (93.9 - 96.0)	63.7% (61.7 - 65.6)	77.8% (76.4 - 79.2)	31.7% (29.7 - 33.6)	0.886 (0.875 - 0.895)
FNA	42.7% (40.3 - 45.0)	73.6% (71.8 - 75.4)	59.7% (58.1 - 61.3)	42.9% (40.5 - 45.4)	0.582 (0.566 - 0.597)
FNA^#^	43.1% (40.6 - 45.5)	81.4% (79.7 - 83.0)	64.1% (62.6 - 65.7)	34.4% (31.5 - 37.0)	0.623 (0.607 to 0.638)
P*	0.410	0.000	0.000	0.000	< 0.0001
C-TIRADS					
US	92.6% (91.4 - 93.8)	65.8% (64.0 - 68.0)	77.9% (76.6 - 79.3)	30.9% (29.1 - 32.8)	0.862 (0.850 - 0.872)
FNA	39.9% (37.6 - 42.1)	74.3% (72.5 - 76.1)	58.7% (57.2 - 60.3)	43.9% (41.1 - 46.7)	0.571 (0.555 - 0.586)
FNA^#^	41.3% (39.0 - 43.5)	65.8% (63.8 - 67.8)	54.8% (53.2 - 56.3)	50.1% (47.4 - 52.9)	0.536 (0.520 to 0.551)
P*	0.021	0.000	0.000	0.001	< 0.0001
ATA guideline					
US	96.3% (95.3 - 97.1)	59.9% (57.9 - 61.9)	76.3% (74.9 - 77.6)	33.6% (31.9 - 35.4)	0.868 (0.857 - 0.879)
FNA	42.9% (40.5 - 45.1)	68.8% (66.9 - 70.7)	57.1% (55.5 - 58.8)	46.9% (44.4 - 49.4)	0.559 (0.543 - 0.574)
FNA^#^	41.9% (39.6 - 44.1)	60.3% (58.3 - 62.3)	52.0% (50.5 - 53.6)	53.5% (50.9 - 55.9)	0.511 (0.496 to 0.527)
P*	0.097	0.000	0.000	0.000	< 0.0001
EU-TIRADS					
US	97.1% (96.3 - 97.9)	47.9% (45.8 - 50.0)	70.2% (68.6 - 71.6)	39.4% (37.6 - 41.2)	0.833 (0.821 - 0.845)
FNA	44.9% (42.5 - 47.1)	54.2% (52.1 - 56.3)	50.0% (48.5 - 51.6)	55.4% (53.2 - 57.6)	0.505 (0.489 - 0.520)
FNA^#^	41.7% (39.4 - 44.0)	51.6% (49.6 - 53.7)	47.1% (45.6 - 48.7)	58.5% (56.4 - 60.8)	0.533 (0.518 to 0.549)
P*	0.000	0.000	0.000	0.059	< 0.0001

Data in parentheses are 95% confidence intervals. UFR, unnecessary FNA rate; AUC, area under the curve; US, according to the US-based final assessment; FNA, according to the ACR-TIRADS’s thresholds for FNA; FNA^#^, according to the inherent thresholds for FNA of Kwak-TIRADS, C-TIRADS, ATA guideline, and EU-TIRADS, respectively; EU-TIRADS, European Thyroid Radiology Thyroid Imaging Reporting and Data System; ATA guideline, American Thyroid Association guideline; ACR-TIRADS, American College of Radiology Thyroid Imaging Reporting and Data System; AI-TIRADS, Artificial Intelligence Thyroid Imaging Reporting and Data System; C-TIRADS, Chinese Thyroid Imaging Reporting and Data System; Kwak-TIRADS, Thyroid Imaging Reporting and Data System was issued by Kwak et al; P*, comparison between the FNA and O-FNA.

The six RSSs showed a nuanced difference in diagnostic performance. The highest AUC, which was seen in Kwak-TIRADS, differed little from that of AI-TIRADS (0.886 and 0.883, *P* = 0.178). Significantly lowest specificity and accuracy, along with highest unnecessary FNA rates were seen in EU-TIRADS (47.9%, 70.2% and 39.4%, respectively, all *P* < 0.05). The highest specificity and lowest unnecessary FNA rates were observed in C-TIRADS (65.8% and 30.9%) and without significant differences to that of AI-TIRADS and Kwak-TIRADS (65.8% vs 63.9% vs 63.7%, 30.9% vs 31.5% vs 31.7%, all *P* > 0.05).

### 3.4 Comparison of the diagnostic performance after applying size thresholds of FNA (according to indications for FNA)

In this study, to exclude the possibility that the size threshold of FNA might affect diagnostic performances and unnecessary FNA rates, we used the unified size thresholds for biopsy proposed by ACR-TIRADS to compare it among the six RSSs. **Table 3** summarizes the diagnostic performances of the FNA criteria of the six RSSs. The six RSSs also show a small difference in diagnostic performance. The EU-TIRADS (44.9%) had the highest sensitivity which was similar to that of the ACR-TIRADS, AI-TIRADS, Kwak-TIRADS, and ATA guidelines (41.9%, 42.3%, 42.7%, 42.9%, respectively, all *P* > 0.05). The AI-TIRADS had the highest accuracy (61.9%) and lowest unnecessary FNA rate (38.6%), which were similar to that of the Kwak-TIRADS (59.7% and 42.9%, all *P* > 0.05), C-TIRADS (58.7% and 43.9%, all *P* > 0.05). Significantly lowest accuracy and AUC, along with the highest unnecessary FNA rate were seen in EU TIRADS (50.0%, 0.505, and 55.4%, respectively, all *P* < 0.05).

The three RSSs (EU-TIRADS, C-TIRADS, and ATA guidelines) which incorporated the ACR-TIRADS’ thresholds for FNA showed better diagnostic performance with a specificity of 54.2%, 74.3%, 68.8%, and accuracy of 50.0%, 58.7%, 57.1% compared to 51.6%, 65.8%, 60.3% and 47.1%, 54.8%, 52.0%, respectively, for the original RSSs (which incorporated inherent thresholds for FNA of themselves) (*P* < 0.05 for all). By contrast, the original Kwak-TIRADS showed a higher specificity of 81.4%, accuracy of 64.1%, and a lower unnecessary FNA rate of 34.4% compared to 73.6%, 59.7%, 42.9%, respectively, for Kwak-TIRADS incorporated the ACR-TIRADS’ thresholds for FNA (*P* < 0.05 for all).

## 4 Discussion

To determine if the categorization methods based on US features in each RSS would influence diagnostic performance and unnecessary FNA rates, we compared the diagnostic performance and unnecessary FNA rates of six RSSs (two pattern-based RSSs, two score-based counting method RSSs, and two score-based weighting method RSSs) according to the same size thresholds proposed by ACR TIRADS and US-based final assessment categories, respectively. The diagnostic performances and unnecessary FNA rates of the six RSS were closely comparable, except for the specificity, accuracy and unnecessary FNA rates in the EU-TIRADS. These results suggested that the categorization methods of RSS were not the decisive factors that influenced the diagnostic performances and unnecessary FNA rates. The findings provide evidence for selecting the optimal RSS and building future RSS for thyroid nodule management.

A number of scientific societies have proposed RSS to stratify malignancy risks (2, 11, 13, 14, 16, 21), but no adequate standardized solution has come out. With a wide spectrum of diagnostic performances, these RSSs were shown to be possibly influenced by the thresholds for FNA, the categorization methods of RSS, and the US criteria for nodule classification. Our results indicated that the categorization methods of RSS were not the decisive factors that influenced the diagnostic performances and unnecessary FNA rates. In this work, the diagnostic performances and unnecessary FNA rates of the six RSS were closely comparable, except for the specificity, accuracy and unnecessary FNA rates in the EU-TIRADS. The EU-TIRADS has lowest accuracy, AUC and highest unnecessary FNA rate than other RSSs before and after applying size thresholds of FNA. The EU-TIRADS proposed a pattern-based system defining four categories. The most noteworthy difference with some of the other RSSs is that four highly suspicious features, even if present isolatedly, could define a nodule as being at the highest risk of cancer (EU‐TIRADS 5) without the need of referencing other US features. However, intermediate risk (EU-TIRADS 4) has no features of high suspicion, a finding which may lead to the results of the EU-TIRADS in this study. Further support for this paper’s conclusion was obtained by between-group comparison in the same categorization method. There was no difference in diagnostic performances for the Kwak-TIRADS in comparison to the C-TIRADS. Whereas the AI-TIRADS had, although small, higher specificity, accuracy, AUC, and lower unnecessary FNA rate compared with the ACR-TIRADS, which was very similar to the results reported by Liu et al. (29). Furthermore, there is some previous studies supporting this conclusion. The diagnostic performances of various RSSs were comparable according to the same size threshold for biopsy (8, 24) and the classified categories (27). In the establishment process of the C-TIRADS, the prediction models based on the score-based weighting and counting were constructed, respectively, the AUCs were similar (16).

However, this outcome is contrary to that of J.H.Yoon et al. (20), who found diagnostic performances and unnecessary FNA rates had a tendency between pattern-based RSSs and score-based RSSs. This discrepancy could be attributed to the different samples and methods of diagnostic performance calculated. Thyroid nodules in J.H.Yoon et al.’s study were all 10 mm or larger, while in our study, the percent of nodules sizes ≥10 mm was 56.5% (2229/3944) and sized <10 mm was 43.5% (1715/3944). Furthermore, in their study, the diagnostic performances were calculated using different (inherent) size thresholds for biopsy, which differed from the same size thresholds in our study. Similarly, a recent study indicated that the Kwak-TIRADS incorporating the size thresholds for FNA of ACR-TIRADS showed higher diagnostic performance and a lower unnecessary FNA rate than the original Kwak-TIRADS (17). These results differ from those of our study, which showed higher specificity, accuracy, AUC, and lower unnecessary FNA rate in the original Kwak-TIRADS, compared to the Kwak-TIRADS which incorporated the ACR-TIRADS’ size thresholds (81.4% vs 73.6%, 64.1% vs 59.7%, 0.62 vs 0.58, 34.4% vs 42.9%, all *P* < 0.05). However, this cannot be explained by the size cut-offs for biopsy. Instead, the number of nodules and their size distribution in each category in the enrolled sample could be a more possible explanation (30). Further work is needed to test this conjecture.

Clinicians should choose a more straightforward categorization method of RSS that can be applied easily. The pattern-based RSSs (ATA guidelines, EU-TIRADS et al.) are more intuitive and make reaching a final assessment category much easier, but they may appear complex to an inexperienced radiologist (31) and may not be appropriate for all nodules (8, 9, 32). In previous studies, about 3.4 - 13.9% of nodules did not meet the criteria for any pattern in the ATA guideline (4, 6, 25) and 8.1% (321/3944) in our cohort. However, score-based RSS is suitable for all of the nodules (17, 20, 21, 32). The score-based weighting RSSs (ACR-TIRADS and AI-TIRADS) require radiologists to interpret and assign morphologic categories with close attention (11). In contrast, the score-based counting method RSSs (C-TIRADS and Kwak-TIRADS) have been proven to be practical and easily applicable (33, 34) because the number of suspicious US features was summed without considering that each feature had a different likelihood of malignancy in the counting method RSS. To sum up, the score-based counting method RSS was optimal for daily clinical practice.

A large and growing body of literature has indicated that the differences in diagnostic performance among RSSs are mainly attributed to the variations in the size threshold for biopsy. Most researchers have revealed that the high specificity and low rate of unnecessary biopsies resulted from the larger size cutoffs (8, 17). In contrast, when the nodules smaller than 1 cm in the highest suspicious category were additionally recommended for biopsying in pediatric populations (28), the sensitivity was improved and the specificity was decreased without changing the unnecessary biopsy rate. Thus, it is necessary to select a balanced threshold depending on the clinical situation for the management of thyroid nodules. But, of course, better management of thyroid nodules is required to improve the overall diagnostic performance. The developments in ultrasound imaging technology (such as computer-aided diagnosis based on artificial intelligence, US-elastography, and contrast-enhanced ultrasound) may provide an opportunity to increase the overall diagnostic performance (35–37).

A number of limitations need to be noted regarding the present study. Firstly, This series included only the patients who underwent surgery and FNA in a tertiary referral center. Thus, the proportion of malignant nodules in our study was higher (45.2%) than that in other studies (range, 10.3%-25.8%) (8, 17, 18, 38). Secondly, our study included a large number of cases confirmed by surgical pathology, which may lead to selection bias and a few false negative rates and false positive rates. However, the surgeon will flag the specimen for the pathologist (one largest or/and highest suspicious thyroid nodule was flagged per thyroid lobe), which could minimize the false negative rate and false positive rate. Thirdly, to assess diagnostic performance in real practice, we categorized unclassified nodules based on the ATA guideline as intermediate-suspicion nodules. Although most previous studies have employed this strategy (9, 19, 24), a few haven’t (4, 5). Finally, when calculating diagnostic performance, triages of the six RSSs were dichotomized according to the level of suspicion each assessment category represents. Our results may have differed according to the arbitrary cutoff of each RSS.

## 5 Conclusion

The categorization method used by each RSS was not a determinant in influencing diagnostic performance and unnecessary FNA rate. For daily clinical practice, the score-based counting RSS was an optimal choice. The present study contributes to the selection of an optimal RSS, which provides helpful evidence for constructing future RSS in the management of thyroid nodules. However, more work needed to be done to determine the appropriate size criteria for FNA.

## Data availability statement

The raw data supporting the conclusions of this article will be made available by the authors, without undue reservation.

## Ethics statement

The studies involving human participants were reviewed and approved by the scientific research and clinical trials ethics committee of the First Affiliated Hospital of Zhengzhou University of China. Written informed consent from the participants’ legal guardian/next of kin was not required to participate in this study in accordance with the national legislation and the institutional requirements.

## Author contributions

CF had the conception and design of this study. CF and YC provided the study materials and patients. JL and JY reviewed and analyzed the imaging data. YW and CS performed the statistical analysis. KC provided basic information of all cases. CF wrote the manuscript. All authors contributed to the article and approved the submitted version.
